# A 3D printed electronic wearable device to generate vertical, horizontal and phono-articulatory jaw movement parameters: A concept implementation

**DOI:** 10.1371/journal.pone.0290497

**Published:** 2023-09-13

**Authors:** Taseef Hasan Farook, Saif Ahmed, Md Shoriful Islam Talukder, James Dudley

**Affiliations:** 1 Adelaide Dental School, University of Adelaide, Adelaide, SA, Australia; 2 Department of Electrical and Computer Engineering, North South University, Dhaka, Bangladesh; University of Minnesota School of Dentistry, UNITED STATES

## Abstract

**Purpose:**

The current research aimed to develop a concept open-source 3D printable, electronic wearable head gear to record jaw movement parameters.

**Materials & methods:**

A 3D printed wearable device was designed and manufactured then fitted with open-source sensors to record vertical, horizontal and phono-articulatory jaw motions. Mean deviation and relative error were measured invitro. The device was implemented on two volunteers for the parameters of maximum anterior protrusion (MAP), maximum lateral excursion (MLE), normal (NMO), and maximum (MMO) mouth opening and fricative phono-articulation. Raw data was normalized using z-score and root mean squared error (RMSE) values were used to evaluate relative differences in readings across the two participants.

**Results:**

RMSE differences across the left and right piezoresistive sensors demonstrated near similar bilateral movements during normal (0.12) and maximal mouth (0.09) opening for participant 1, while varying greatly for participant 2 (0.25 and 0.14, respectively). There were larger differences in RMSE during accelerometric motion in different axes for MAP, MLE and Fricatives.

**Conclusion:**

The current implementation demonstrated that a 3D printed electronic wearable device with open-source sensor technology can record horizontal, vertical, and phono-articulatory maxillomandibular movements in two participants. However, future efforts must be made to overcome the limitations documented within the current experiment.

## Introduction

Jaw motion is patient specific and adversely affected by various conditions such as disc derangement and osteoarthritis which cannot be simulated in vitro [1]. Various methods are available for analyzing patient-specific jaw relationships. One of them is the manual application of facebow articulators where the technician mounts the patient’s maxillary and mandibular models on an articulator to reproduce the patient’s jaw movements [2]. Such methods are prone to subjective biases as evaluation criteria differ across practitioners [3]. Axiography measures the movements of the mandible with respect to the cranium using strain gauge or linear variable differential transformers [4]. Electromyography measures the electrical activity of the muscles involved in jaw movement. Electrognathography and Optoelectronic oral or extra-oral tracking capture the movement of the jaw in three-dimensional space using markers placed on the patient’s face or head with a combination of infrared and magnetic sensors [2, 4].

The methods of diagnostic analysis vary in their approaches and technologies, and each possesses unique advantages and limitations depending on the patient’s requirements and the research or clinical application. However, the high cost of specialized equipment and maintenance can make it difficult for remote rural practices and developing economies to access them [5]. Additionally, most primary care practices may not require such complex and expensive equipment and would only need basic evaluation tools to aid in estimating whether any side of the jaws are affected, scheduling appropriate referrals, and reducing waiting periods.

The technology for analyzing jaw movement has evolved from heavy and elaborate setups to lightweight wearable prostheses using advanced sensing technologies. In addition, computing has been successful in mitigating most of the variables that previously affected the accuracy of readings, such as mandibular deviation, tooth morphology, and variations in natural anterior guidance [6]. Although not yet utilized for jaw diagnostics, the implementation of open-source technology has fostered greater inclusivity within the research community, particularly in the field of digital dentistry [7].

The key benefits of 3D printing in dentistry include increased accuracy and precision in the fabrication of dental devices, reduced chairside time for patients, and the ability to customize devices for individual patients [8]. 3D printing also allows for the production of complex geometry and the use of novel inexpensive materials without generating much wastage [9]. However, the technology is still relatively new in dentistry with concerns about the durability of some 3D-printed materials, as well as potential regulatory challenges [10]. Such issues can be overcome if the implementation is made open source and organizations are able to improve upon the model to cater to their specific needs.

While several advances in biomedical engineering have been made in the space of jaw movement analysis, the development and concept validation of an open-source, 3D printable, electro-mechanical wearable system is still lacking. The current manuscript aimed to develop a 3D printable electronic wearable head gear to record jaw movement parameters. At the current conceptualization stage, the goal was not to demonstrate accurate clinical diagnostics, but rather develop an open-source custom-fit framework that read various jaw movements and produce quantitative outputs.

## Materials and methods

The current concept evaluation was conducted as a part of a study approved by the University’s Human Research and Ethics Commission (project approval code H-2022-185).

### Device framework construction

**Fig 1:**
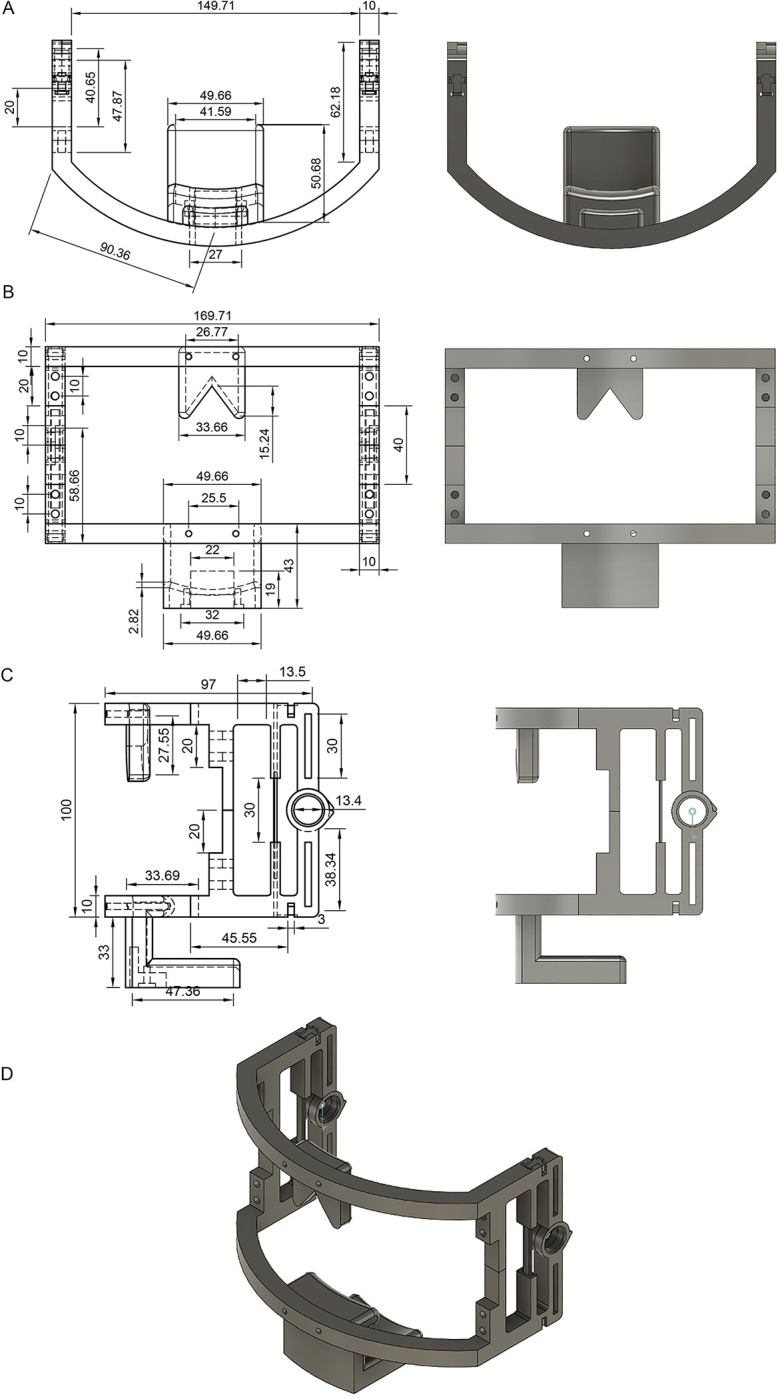
Blueprint schematics (left) and subsequent CAD mockups (right) for the 3D printable jaw movement analysing device **A)** Axial slice, **B)** Coronal slice, **C)** Sagittal slice, **D)** Assembly mockup.

A blueprint schematic of the mechanical component was created, and the prototype device was designed using a computer-aided design (CAD) mockup **(Fig 1).** The files were subsequently 3D printed using fused deposition modelling (Ender-3; Creality Inc., China) using polylactic acid filament. The following parameters were used for the print job: triangular infill pattern with an infill density of 30%, 100% surface density, and a print material weight of 93 grams with no support material and printed at a speed of 60 mm/s.

The wearable device consisted of the following:

1. A maxillary arch with a facial rest extending from the soft tissue nasion to the nasal bridge
2. A mandibular arch with a symphyseal chin rest extending to the menton region resulting in a supramentale attachment via a chin mount. The symphyseal midpoint of the arch housed the accelerometer and gyroscopic sensor (ADXL345; Analogue Devices Inc., USA) which measured mandibular x-axis (horizontal), y-axis (sagittal), and z-axis (protrusive) movements. To account for vertical soft tissue displacement, the chin rest was attached over an area of soft tissue B point to Menton with the use of foam adhesive
3. Two sagittal brackets to connect the maxillary and mandibular arcs, contain the piezoresistive angle sensors that bend to measure resistance produced upon jaw opening and closing (SEN-00024 Flex 2.2”; Sparkfun Electronics, USA), and 13.4mm motion bearings (B017 FAG; Schaeffier Group, Germany) with a lockout mechanism employed at the hinge preventing openings beyond 75mm.
4. Nylon 6/6 polyamide strap cable ties to connect the parts while allowing for freedom of lateral motion without causing stress on the brackets.
5. Elastic bands with hook & loop fastening (SJ3401; 3M, USA) for attachment to the back of the head functioning to encourage retruded contact position (RCP). Ensuring the RCP is essential for most restorative and rehabilitative dental procedures as it is the position where bites need to be registered. An abnormal RCP bite after mechanical facilitation using the elastic bands can indicate temporomandibular joint disorders or occlusal disharmony.

**Fig 2:**
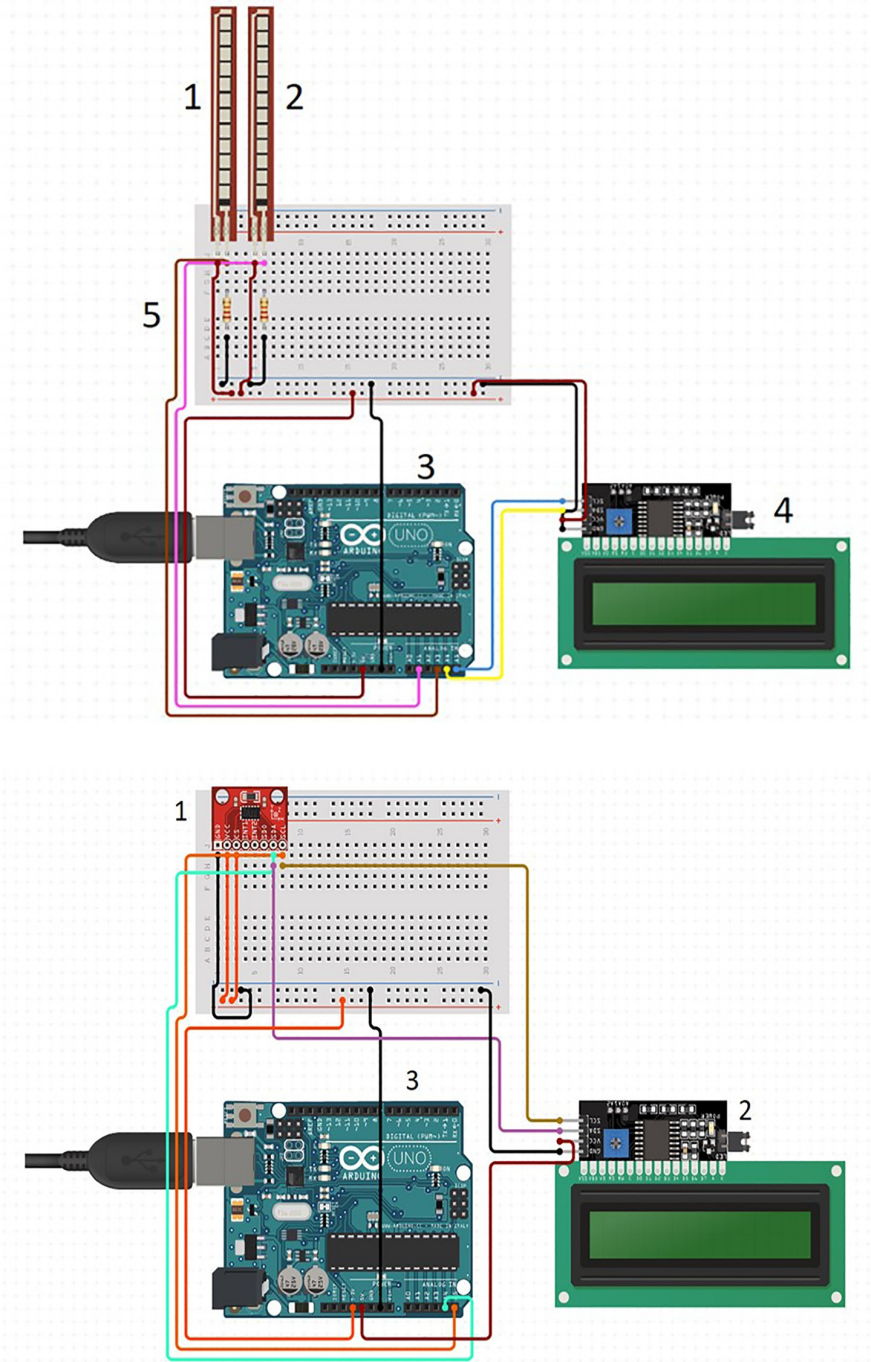
Sensor schematics. A. Jaw movement sensor schematics for displacement analysis. (1 & 2 = piezoresistive angle sensors, 3 = Arduino Uno, 4 = I2C 1602 LCD, 5 = Resistor 10K). B. Jaw movement sensor schematics for accelerometric analyses. (1 = Accelerometer sensor, 2 = I2C 1602 LCD, 3 = Arduino Uno).

The circuit diagram for the electronic components have been provided in **Fig 2A** and **2B.**

### Device testing

**Fig 3:**
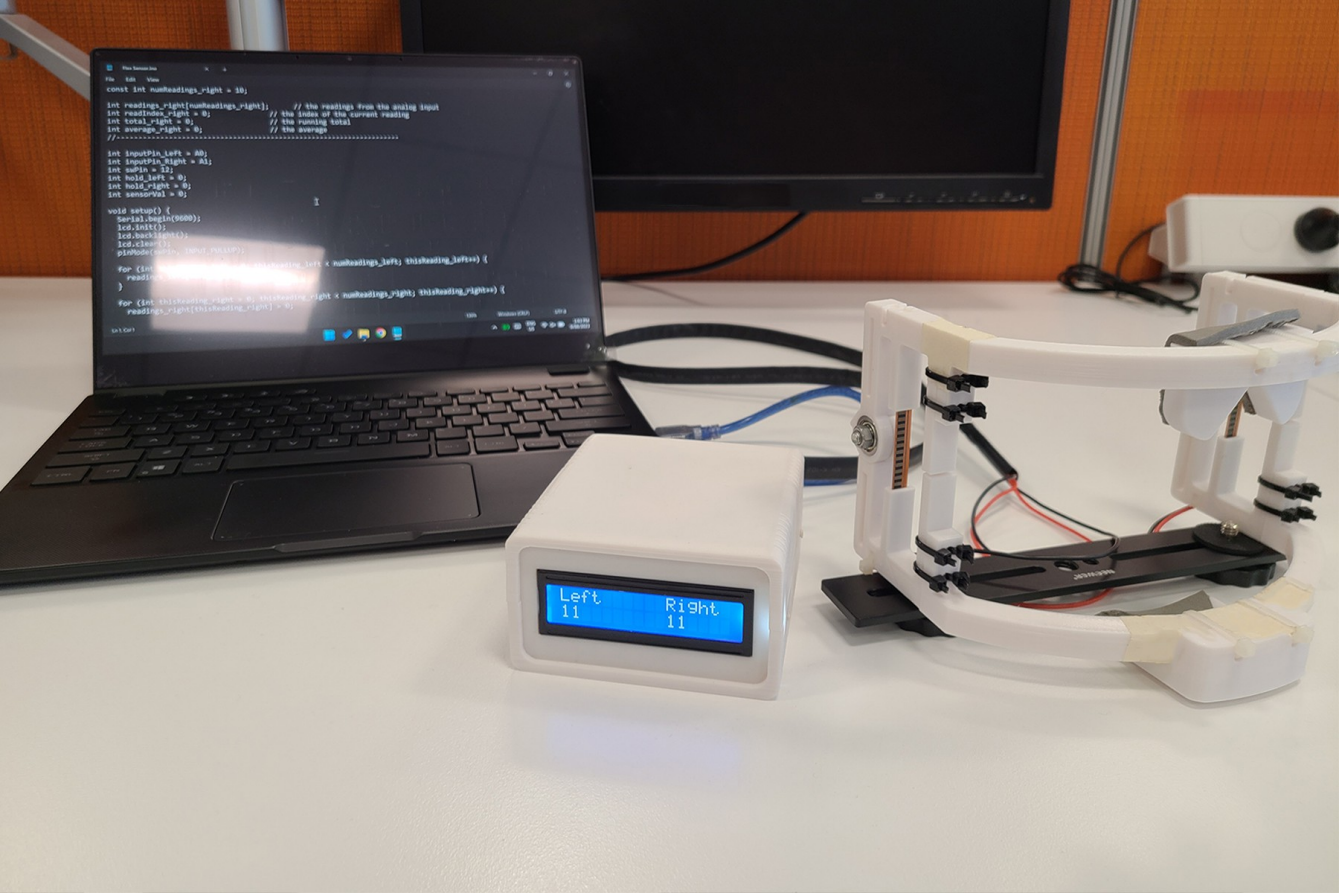
Stabilization and tabletop calibration testing.

In vitro tabletop testing was first performed to evaluate the absolute error margins produced by the device on a vertical plane to simulate opening without having to factor in biological variations. The measurements were carried out by stabilizing the headgear on a platform and vertically displacing the arches from the center into eight open positions ranging from partial opening to maximum opening **(Fig 3).** The output values were compared to physical displacements measured via electronic calipers. The following formula were employed for the purpose:

•Absoluteerror=measuredvalue−actualvalue

An in vivo calibration analysis or evaluation of relative error margins in the current stage was deemed impractical owing to the numerous biological and habitual variations that would need to be considered and only possible when calibrated across a large participant cohort.

Following prototype finalization and sensor calibration on 20th October 2022, two conveniently selected adult volunteers provided consent for a single session of evaluation with no specific screening criteria applied. Participant 1, a 26-year-old male with a mean incisal opening of approximately 35 mm otherwise medically fit and well, and Participant 2, a 27-year-old female with a mean incisal opening of less than 25 mm, no history of trauma, and undergoing medical treatment for osteoarthritis of multiple joints, were the subjects of the study. As the purpose of the experiment was to serve as a functionality test for the device as opposed to diagnosing the individuals, no identifiable information, medical records, or dental screening other than the ones stated above were collected.

**Fig 4:**
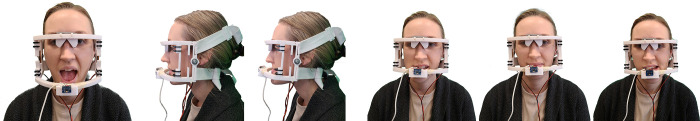
Application of wearable device. A. Jaw Opening. B. Maximum anterior protrusion. C. Maximum lateral deviation.

Both participants were instructed on the maneuvers and actions required to produce readings for normal mouth opening (NMO) **(Fig 4A)**, maximum mouth opening (MMO), maximum anterior protrusion (MAP) **(Fig 4B)**, maximum lateral excursion (MLE) **(Fig 4C)**, and fricative phono-articulations. The two participants repeated each movement for 5 seconds based on a previous report of digital analyses of jaw movement parameters [11]. To determine phono-articulatory competence, the participants were asked to say, “father found some coffee”, as established to be the benchmark for fricative evaluation in a historic investigation [12]. To account for procedural errors, the participants were asked to repeat all the actions five times which were unrecorded, and movements corrected by the operator if required. It was during this time that the operators also ensured repeatability of the data generated. After each action, the sensors were returned to baseline using a physical reset switch on the microcontrollers (Arduino UNO; Smart Projects, Italy) regulating the data capture. The codes applied for the purpose of data collection have been documented in **the S1 File**. Readings were recorded from the sixth set of actions onwards for both participants.

**Fig 5:**
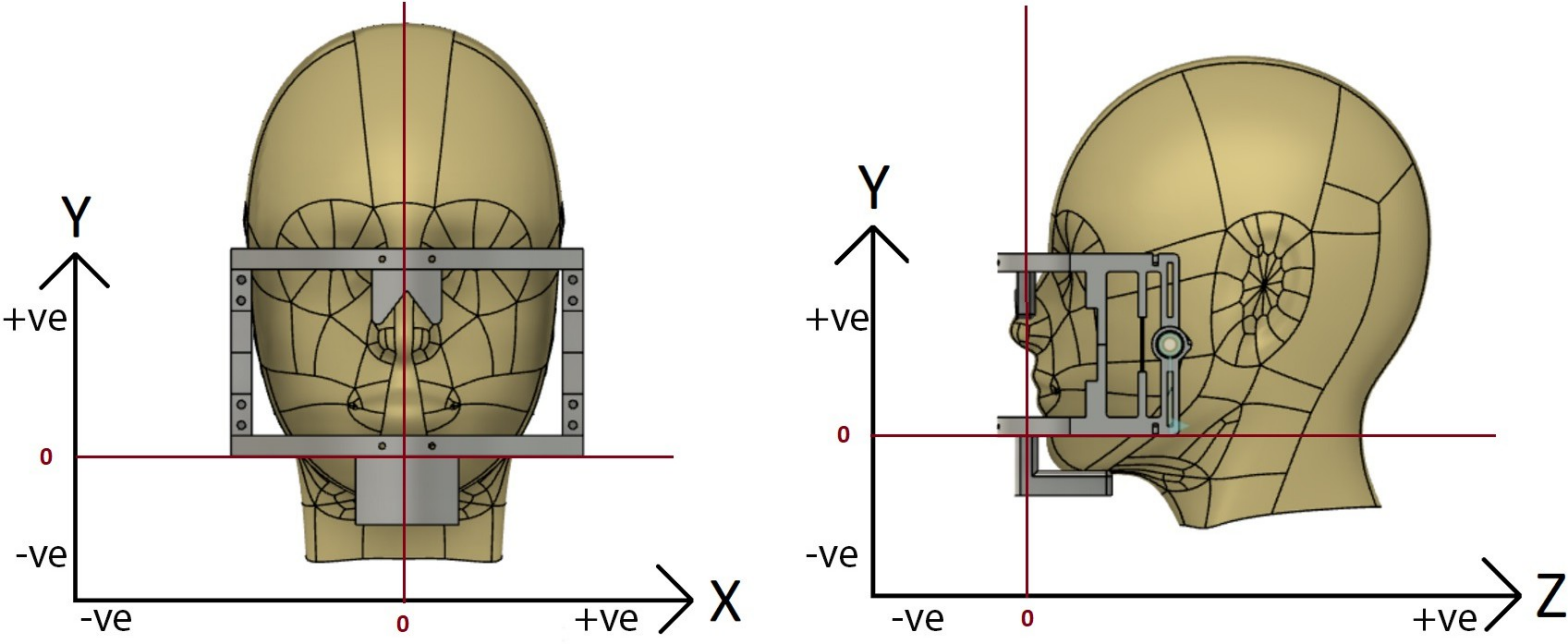
Jaw movement directions respective to the values obtained across the accelerometer-gyroscope sensor axes (X, Y, and Z axes).

The lateral and sagittal axes of movement have been demonstrated in **Fig 5**. Data normalization was introduced to remove inherent biases introduced by variations in sensor sensitivity or individual biological variations between the participants [13]. For the purpose, z-scores and root mean squared error values were calculated for the raw sensor values using the following formulae:

$$•\;Z=\frac{\left(X-\mu \right)}{\sigma }$$

Here Z represents the z-score, also known as the standard score, X represents a specific data point or value from the dataset, μ is the mean of the dataset and σ is the standard deviation of the dataset.


$$•\;RMSE=\sqrt{\frac{\sum {\left(A_{i}-B_{i}\right)}^{2}}{N}}$$

Here RMSE represents the root mean squared error, *A_i_* and *B_i_* are gyroscope results for each participant and *N* is the number of data points being used.

## Results

**Fig 6:**
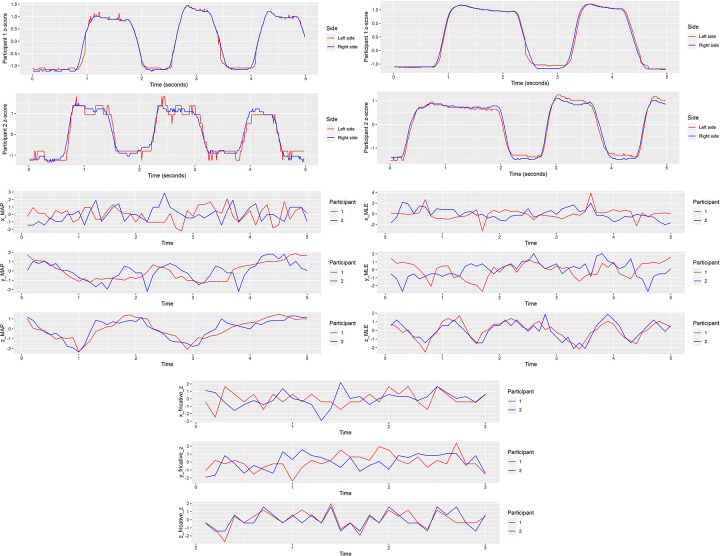
Normalization and visualization of sensor readings across the two participants. A. z-score values for left and right sided piezoresistive sensor values for normal mouth opening (x axis = time in seconds). B. z-score values for left and right sided piezoresistive sensor values for maximum mouth opening (x axis = time in seconds). C. z-score values for accelerometric motion in x, y, and z axes during maximum anterior protrusion (x axis = time in seconds). D. z-score values for accelerometric motion in x, y, and z axes during maximum lateral excursion (x axis = time in seconds). E. z-score values for accelerometric motion in x, y, and z axes during fricative phonetics (x axis = time in seconds).

Normal mouth-opening cycle differences were estimated from readings generated by electro-resistive sensors on the left and right side. Absolute errors across eight in vitro readings were seen to be 0.87 ±1.67mm. Outcomes of normalization using z-score have been illustrated for NMO **(Fig 6A)**, MMO **(Fig 6B)**, MAP **(Fig 6C)**, MLE **(Fig 6D)**, and Fricatives **(Fig 6E)**. The smaller differences in RMSE across the left and right piezoresistive sensors demonstrated near similar bilateral movements during normal (0.12) and maximal mouth (0.09) opening for participant 1, while varying greatly for participant 2 (0.25 and 0.14, respectively). Alternatively, the larger differences in RMSE during accelerometric motion in MAP, MLE and Fricatives **(Table 1)**, while inconclusive owing to a lack of a larger sample data, is suggestive of substantial differences displayed across the two participants [14].

**Table 1:** RMSE values for accelerometer sensor data outputs.

	MAP	MLE	Fricative phonetics
**x_axis**	1.51	1.36	1.42
**y_axis**	0.93	1.52	1.31
**z_axis**	0.53	0.64	0.68

## Discussion

The current manuscript aimed to develop and validate a 3D printable electronic wearable device to record relative jaw movement data. Mouth opening, protrusion, and lateral excursions were categorized as the most important evaluation criteria in the validation of mandibular motion trajectory tracking systems and were therefore evaluated in the current experiment [15].

Inflammatory disorders such as osteoarthritis can restrict bony translation as well as generate resistive muscle tension [16]. Historically, drastic changes in jaw movement have been reportedly caused by a shallow compensating curve, shortened dental arch, trauma, TMD and severe changes to occlusal facets, condylar guidance and certain neuroendocrine conditions [17, 18]. The current study found participant 2 to have shorter quantitative maximum mouth opening cycles, leading to more frequent movement cycles per 5 seconds, consistent with their clinical presentations while normal mouth opening cycles were seemingly unaffected. This is supported by the finding that pressure pain threshold for patients with inflammatory or degenerative disorders such as osteoarthritis is lower, thus leading to significantly lower jaw opening distances [19]. The current report is however contradicted by the report that osteoarthritis leads to slower jaw closing velocity, that would imply that fewer opening and closing cycles should be recorded per time [19].

Virtual articulators, 3D scanners and radiomics-guided jaw tracing are expensive, not readily available, and possess their own disadvantages [2]. An alternative cost-effective approach would be to video record jaw movement and mark the regions and landmarks of interest within each image frame. The current investigation did not use operator-labelled image-based tracking to mark jaw motion as a recent report found extreme variations to interrater reliability (0.13 to 0.65) and very low confidence intervals (< 0.6) [20]. Furthermore, any outcomes generated from datasets annotated by practitioners are greatly affected by their clinical experience [21]. However, with the advent of unsupervised deep learning [22], real-time landmark recognition [23], and explainable learning [24], future attempts may be performed to integrate open-source optoelectronic tracking to record intricate jaw movement readings.

While most acceleration values can be predicted with a linear trendline, fluctuations in z-score values were noted and can be owed to a possible lack of sensor linearity and internal deterministic errors within the sensor itself [25]. Aside from occlusal irregularities, a high y-axis movement could also be accredited to occasional backward tilts of the head during specific functions such as jaw protrusion and the subsequent residual state of open mouth that follows [26]. Although the physiological phenomenon is considered normal, to further standardize data outputs, sensors to counterbalance head movement will be implemented in the next iteration with to offset unwanted skull movements and improve stability of readings during extensive head tilting.

The current device design did not use an intraoral stabilization component which can be considered both an advantage and limitation. Previously, investigators found intraoral mandibular stabilization units to disturb physiological salivary functions and were stated to alter mandibular motion [16]. The supramentale attachment and menton rest in the current study was designed to mitigate most of the influence that the soft tissue might pose during the jaw motions. That said, soft tissue movement has always affected jaw movement analyzing devices [4] and can serve as a source of variation that can be corrected in the future with deep learning-based interventions [27, 28]. Additionally, standard intraoral attachments are not ideal for fricative phono-articulation as the incisors need to make contact with the lower lip and produce a seal, which is impeded by the extraoral extension from the stabilization trays. To enhance the current design of the wearable device, several modifications can be made. These include creating a thinner chassis, increasing modularity to accommodate varying head sizes, and installing fiducial markers to enable easy tracking through various object detection algorithms for real-time image tracing [5, 29]. The use of image tracing has enabled accurate dental diagnoses even with low-resolution, noise-prone imaging [30], which suggests that low-cost cameras may be a viable option for this purpose. By incorporating such changes, the device’s readings can be more detailed, making it possible to apply deep learning-based predictive models like reinforcement learning. These models can establish trends in jaw motion and predict anomalies by referring back to a database founded on a trial-and-error model applied across a large participant pool of varying jaw movement parameters.

The variations in x and y axes were exacerbated in participant 2 who reduced jaw movement during fricative speech and could be accredited to disturbed dimensionality of jaw orientations [31]. Another consideration should be towards challenges faced by individuals with their English accent and pronunciation during fricative phonetics and could have accounted for some of the fluctuations. This is deemed relevant amidst the knowledge that phonetic articulation evaluations are not linguistically universal and individuals may demonstrate hesitation in use, accented speech variations, and truncations in a language that is not native to them [32, 33].

Lateral excursions of the jaw were regulated by cabled attachment cords with some elastic slack. Future revisions in the design can incorporate the development of reusable, highly viscoelastic straps with low modulus of elasticity to connect the pieces of the device together and facilitate completely natural lateral excursions without providing resistance in the physiological motion and dynamics to improve versatility but keep the overall design cost effective.

### Limitations

The current design has several limitations. Friction at the moving mechanical parts produced minute resistances which needed to be measured, while aftermarket sensor sensitivity varies among manufacturers and must be pre-calibrated. Therefore, the current sensor calibration may not apply universally as the physical device fabrication process is always associated with mismatches of material thickness, length, and material density. Therefore, comparative calibrations using a goniometer to correlate true displacement in millimeters from the sensor data were not performed. Instead, the outputs were presented in their raw original units, that is resistive ohms or accelerometric microns/s. This was also because realistically unavoidable variations like environmental resistance, friction and minute physical movements requires calibration across a larger dataset to standardize the sensor readings into meaningful measurements useful to the clinician. Therefore, instead of producing absolute parameters, the current study implemented normalization analyses on the relative measurements to validate whether the device is in fact capable of generating data for different jaw movement conditions using open-source sensor outputs. That said, the anatomical structure differences of the participants’ face and jaw were not considered during the evaluation and can lead to biases when reflected on a larger data pool and can be mitigated by increased modularity of the framework. Health condition differences, speaker idiosyncrasies and biological conditions leading to poor device fit could further influence observed differences in jaw movement parameters. The amount of time spent on completing specific functions also varied. Therefore, a detailed participant history and a thoroughly rehearsed set of instructions will be required during future diagnostic evaluations, that too on larger participant pools to establish reliability. Finally, deflections and deviations during mouth opening can affect bilateral vertical displacement and cannot be quantified within the current setup without having additional sensors to measure horizontal displacement chances.

Piezoresistive sensors are temperature dependent, and discharge stored current when retained within a specific angle for prolonged duration. This would indicate that prolonged periods of open jaw positions would cause substantial current discharge and a gradual decrease in value output unless recalibrated at every instance. The current study was unaffected in said regard as the motions were of basic opening and closing without delays between the two processes. Therefore, future experiments that require prolonged periods of mouth opening or needing data from minute movements should replace the existing angle sensors with either linear displacement or contactless position sensors, or a complete overhaul with lidar sensors. Accelerometer sensors were originally designed to measure non-quantitative direction of motion and produce data specific to the individual sensor’s factory calibration. Therefore, data conversion to displacement, in millimeters, requires repeated zero calibration and measurements of acceleration and velocity in the axis of motion. Future studies will include incorporate image-based object detection, repeated calculations of velocity, displacement, and time across a range of TMJ movements using accelerometers from different manufacturers to generate a universal calibration workflow to make these sensors immediately usable in clinical jaw movement analysis. Once fully calibrated, the device will be tested on a larger sample size. Overall, 3D printing shows promise as a scalable tool for feasibly advancing the field of digital dentistry, but continued collaborative research and development is needed to fully realize its potential in the complicated subspecialty of jaw movement and occlusal analyses.

## Conclusion

The current experiment confirmed that a 3D printed electronic wearable device with open-source sensor technology can record horizontal, vertical, and phono-articulatory maxillomandibular movements in two subjects. However, modifications are required to overcome the mentioned limitations.

## Supporting information

S1 Checklist STROBE statement—checklist of items that should be included in reports of observational studies. (DOCX)

S1 File (PDF)
