# Supplementary material for: A 3D printed electronic wearable device to generate vertical, horizontal and phono-articulatory jaw movement parameters: A concept implementation
Source: PLoS One. 2023 Sep 13;18(9):e0290497. doi: 10.1371/journal.pone.0290497 (PMC10499219; doi:10.1371/journal.pone.0290497)
Supplement: S1 File — (PDF) [file pone.0290497.s002.pdf]

A 3D printed electronic wearable device to generate vertical, horizontal and phono-articulatory jaw movement parameters: a concept implementation

SUPPLEMENTARY DOCUMENT

Codes to generate accelerometer data output.

```
#include <Wire.h>
#include <ADXL345.h>
#include <LiquidCrystal_I2C.h>

LiquidCrystal_I2C lcd(0x27, 16, 2);


ADXL345 adxl; //variable adxl is an instance of the ADXL345 library

void setup() {
  Serial.begin(9600);
  lcd.init();
  lcd.backlight();
  adxl.powerOn();

  //set activity/ inactivity thresholds (0-255)
  adxl.setActivityThreshold(75); //62.5mg per increment
  adxl.setInactivityThreshold(75); //62.5mg per increment
  adxl.setTimeInactivity(10); // how many seconds of no activity is inactive?

  //look of activity movement on this axes - 1 == on; 0 == off
  adxl.setActivityX(1);
  adxl.setActivityY(1);
  adxl.setActivityZ(1);

  //look of inactivity movement on this axes - 1 == on; 0 == off
  adxl.setInactivityX(1);
  adxl.setInactivityY(1);
  adxl.setInactivityZ(1);

  //look of tap movement on this axes - 1 == on; 0 == off
  adxl.setTapDetectionOnX(0);
  adxl.setTapDetectionOnY(0);
  adxl.setTapDetectionOnZ(1);

  //set values for what is a tap, and what is a double tap (0-255)
  adxl.setTapThreshold(50); //62.5mg per increment
  adxl.setTapDuration(15); //625us per increment
  adxl.setDoubleTapLatency(80); //1.25ms per increment
```

```
  adxl.setDoubleTapWindow(200); //1.25ms per increment

  //set values for freefall (0-255)
  adxl.setFreeFallThreshold(7); //(5 - 9) recommended - 62.5mg per increment
  adxl.setFreeFallDuration(45); //(20 - 70) recommended - 5ms per increment

  //set interrupts to take place on int pin 1
  adxl.setInterruptMapping( ADXL345_INT_SINGLE_TAP_BIT,   ADXL345_INT1_PIN );
  adxl.setInterruptMapping( ADXL345_INT_DOUBLE_TAP_BIT,   ADXL345_INT1_PIN );
  adxl.setInterruptMapping( ADXL345_INT_FREE_FALL_BIT,    ADXL345_INT1_PIN );
  adxl.setInterruptMapping( ADXL345_INT_ACTIVITY_BIT,     ADXL345_INT1_PIN );
  adxl.setInterruptMapping( ADXL345_INT_INACTIVITY_BIT,   ADXL345_INT1_PIN );

  //register interrupt actions - 1 == on; 0 == off
  adxl.setInterrupt( ADXL345_INT_SINGLE_TAP_BIT, 1);
  adxl.setInterrupt( ADXL345_INT_DOUBLE_TAP_BIT, 1);
  adxl.setInterrupt( ADXL345_INT_FREE_FALL_BIT,  1);
  adxl.setInterrupt( ADXL345_INT_ACTIVITY_BIT,   1);
  adxl.setInterrupt( ADXL345_INT_INACTIVITY_BIT, 1);
}

void loop() {

  int x, y, z;
  adxl.readXYZ(&x, &y, &z); //read the accelerometer values and store them in variables  x,y,z
  // Output x,y,z values
  Serial.print("values of X , Y , Z: ");
  Serial.print(x);
  Serial.print(" , ");
  Serial.print(y);
  Serial.print(" , ");
  Serial.println(z);

  double xyz[3];
  double ax, ay, az;
  adxl.getAcceleration(xyz);
  ax = xyz[0];
  ay = xyz[1];
  az = xyz[2];
  Serial.print("X=");
  Serial.print(ax);
  Serial.println(" g");
  Serial.print("Y=");
  Serial.print(ay);
  Serial.println(" g");
  Serial.print("Z=");
  Serial.print(az);
  Serial.println(" g");
```

```
  Serial.println("**********************");

  lcd.setCursor(0, 0);
  lcd.print("X");
  lcd.setCursor(0, 1);
  lcd.print(x);
  lcd.setCursor(6, 0);
  lcd.print("Y");
  lcd.setCursor(6, 1);
  lcd.print(y);
  lcd.setCursor(12, 0);
  lcd.print("Z");
  lcd.setCursor(12, 1);
  lcd.print(z);
  delay(500);
  lcd.clear();

}
```

Codes to generate piezoresistive angle sensor data output

```
#include <LiquidCrystal_I2C.h>

LiquidCrystal_I2C lcd(0x27, 16, 2);

// Sensors precalibrated
//-------------------------------------------------------------------
const int numReadings_left = 10;

int readings_left[numReadings_left];      // the readings from the analog input
int readIndex_left = 0;              // the index of the current reading
int total_left = 0;                  // the running total
int average_left = 0;                // the average
//-------------------------------------------------------------------

//-------------------------------------------------------------------
const int numReadings_right = 10;

int readings_right[numReadings_right];      // the readings from the analog input
int readIndex_right = 0;              // the index of the current reading
int total_right = 0;                  // the running total
int average_right = 0;                // the average
//-------------------------------------------------------------------

int inputPin_Left = A0;
int inputPin_Right = A1;
int swPin = 12;
int hold_left = 0;
int hold_right = 0;
int sensorVal = 0;

void setup() {
 Serial.begin(9600);
 lcd.init();
 lcd.backlight();
 lcd.clear();
 pinMode(swPin, INPUT_PULLUP);

 for (int thisReading_left = 0; thisReading_left < numReadings_left; thisReading_left++) {
   readings_left[thisReading_left] = 0;
 }

 for (int thisReading_right = 0; thisReading_right < numReadings_right; thisReading_right++) {
   readings_right[thisReading_right] = 0;

 }
}

void loop() {
 sensorVal = digitalRead(swPin);
```

```
if (sensorVal == HIGH) {

  //-------------------------------------------------------------------
  // subtract the last reading:
  total_left = total_left - readings_left[readIndex_left];
  // read from the sensor:
  readings_left[readIndex_left] = analogRead(inputPin_Left);
  // add the reading to the total:
  total_left = total_left + readings_left[readIndex_left];
  // advance to the next position in the array:
  readIndex_left = readIndex_left + 1;

  // if we're at the end of the array...
  if (readIndex_left >= numReadings_left) {
    // ...wrap around to the beginning:
    readIndex_left = 0;
  }

  // calculate the average:
  average_left = total_left / numReadings_left;
  //-------------------------------------------------------------------


  //-------------------------------------------------------------------
  // subtract the last reading:
  total_right = total_right - readings_right[readIndex_right];
  // read from the sensor:
  readings_right[readIndex_right] = analogRead(inputPin_Right);
  // add the reading to the total:
  total_right = total_right + readings_right[readIndex_right];
  // advance to the next position in the array:
  readIndex_right = readIndex_right + 1;

  // if we're at the end of the array...
  if (readIndex_right >= numReadings_right) {
    // ...wrap around to the beginning:
    readIndex_right = 0;
  }

  // calculate the average:
  average_right = total_right / numReadings_right;
  //-------------------------------------------------------------------


  // send it to the computer as ASCII digits
  Serial.print(average_left);
  Serial.print("\t");
  Serial.println(average_right);
```

```
    lcd.setCursor(0, 0);
    lcd.print("Left");
    lcd.setCursor(0, 1);
    lcd.print((average_left - hold_left));

    lcd.setCursor(10, 0);
    lcd.print("Right");
    lcd.setCursor(10, 1);
    lcd.print((average_right - hold_right));

    delay(1);        // delay in between reads for stability
  }

  else
  {
    hold_left = analogRead(inputPin_Left);
    hold_right = analogRead(inputPin_Right);
    delay(1000);
    lcd.clear();
  }
}
```
